# Hepatocyte nuclear factor 1α downregulates HBV gene expression and replication by activating the NF-κB signaling pathway

**DOI:** 10.1371/journal.pone.0174017

**Published:** 2017-03-20

**Authors:** Junyu Lin, Chenjian Gu, Zhongliang Shen, Yanfeng Liu, Wei Wang, Shuai Tao, Xiaoxian Cui, Jing Liu, Youhua Xie

**Affiliations:** Key Laboratory of Medical Molecular Virology (Ministry of Health and Ministry of Education), Shanghai Medical College, Fudan University, Shanghai, China; Yonsei University, REPUBLIC OF KOREA

## Abstract

The role of hepatocyte nuclear factor 1α (HNF1α) in the regulation of gene expression and replication of hepatitis B virus (HBV) is not fully understood. Previous reports have documented the induction of the expression of viral large surface protein (LHBs) by HNF1α through activating viral Sp1 promoter. Large amount of LHBs can block the secretion of hepatitis B surface antigen (HBsAg). Here we found that *HNF1α* overexpression inhibited HBV gene expression and replication in Huh7 cells, resulting in marked decreases in HBsAg, hepatitis B e antigen (HBeAg) and virion productions. In contrast, knockdown of endogenous *HNF1α* expression enhanced viral gene expression and replication. This HNF1α-mediated inhibition did not depend on LHBs. Instead, HNF1α promoted the expression of *NF-κB p65* and slowed p65 protein degradation, leading to nuclear accumulation of p65 and activation of the NF-κB signaling, which in turn inhibited HBV gene expression and replication. The inhibitor of the NF-κB signaling, IκBα-SR, could abrogate this HNF1α-mediated inhibition. While the dimerization domain of HNF1α was dispensable for the induction of LHBs expression, all the domains of HNF1α was required for the inhibition of HBV gene expression. Our findings identify a novel role of HNF1α in the regulation of HBV gene expression and replication.

## Introduction

The infection of hepatitis B virus (HBV), which affects 240 million people worldwide, can cause acute and chronic liver diseases [1]. HBV is an enveloped virus with a genome of 3.2kb partially double-stranded relaxed circular DNA (rcDNA) within its nucleocapsid. After infection of the hepatocyte, rcDNA is converted in the nucleus to covalently closed circular DNA (cccDNA) that serves as the template for viral transcription. Four promoters (Sp1, Sp2, Cp, and Xp) in concert with two enhancers (EnI, EnII) control HBV transcription. The 3.5kb preC mRNA and pregenomic (pg) RNA encode hepatitis e antigen (HBeAg) and the core and polymerase proteins, respectively. The 2.4kb preS1 mRNA encodes the large surface protein (LHBs) and the 2.1kb preS2/S mRNA the middle and small surface proteins (MHBs & SHBs). The 0.7kb mRNA encodes HBx. HBV polymerase associates with pgRNA, resulting in their encapsidation by core proteins. In the nucleocapsid, pgRNA is reverse transcribed to rcDNA by viral polymerase. New nucleocapsids are enveloped by viral surface proteins and secreted, or traffick rcDNA back to the nucleus to supplement the cccDNA pool [2, 3].

Liver-enriched transcription factors (LETFs) are key factors regulating the expression of many liver-specific genes involved in metabolism, proliferation and differentiation of hepatocytes [4, 5]. Several LETFs can bind to HBV promoters to regulate viral gene expression and replication, including hepatocyte nuclear factor 1α (HNF1α), hepatocyte nuclear factor 3 (HNF3), hepatocyte nuclear factor 4α (HNF4α), peroxisome proliferator-activated receptor α (PPARα), retinoic acid receptor α (RXRα), and CCAAT/enhancer binding protein (C/EBP) [6–10]. HNF1α is composed of an N-terminal dimerization domain [amino acids (aa) 1–32), a POU-homeobox DNA binding domain (aa150-280) and a C-terminal transactivation domain (aa281-631) [11]. By binding to a 13-bp conserved sequence in the Sp1 promoter, HNF1α induces the expression of the preS1 mRNA to promote the synthesis of LHBs [12–14]. Overexpression of LHBs causes the retention of subviral and viral particles in endoplasmic reticulum (ER) [12–14]. HNF1α has been reported to act synergistically with HNF4α [15], Oct1 [16] and LRH-1 [17] in promoting HBV gene transcription. However, HNF1α has also been reported to downregulate HBV replication by repressing EnI [18]. Moreover, HNF1α-null HBV transgenic mice do not display a notable change in the preS1 mRNA level in hepatocytes compared with normal HBV transgenic mice, but manifest a significant increase in viral replication intermediates and cccDNA that is absent in normal HBV transgenic mice [19]. These seemingly conflicting findings suggest a complex role of HNF1α in the regulation of HBV gene expression and replication.

The major form of nuclear factor-kappa B (NF-κB) in hepatocytes is a heterodimeric complex composed of a 50-kDa subunit (p50) and a 65-kDa subunit (p65) [20–22]. Under nonstimulatory conditions, NF-κB is sequestered in the cytoplasm, associated with inhibitory IκB family proteins. Treatment of cells with stimuli such as inflammatory cytokines, bacterial products, viruses or mitogens can lead to phosphorylation of serines 32 and 36 of IκBα or serines 19 and 23 of IκBβ. The phosphorylated IκB proteins undergo rapid ubiquitin-mediated proteasomal degradation, resulting in the release and translocation of NF-κB into the nucleus where NF-κB activates many NF-κB-responsive genes [23, 24]. Activation of the NF-κB signaling is an indicative event during viral infection [25], which induces the expression of numerous genes related to inflammation, antiviral defense and other critical cellular processes such as apoptosis. It is only natural that NF-κB is a common target hijacked by viruses. For HBV, HBx has been reported to induce the phosphorylation of IκBα and activate the NF-κB signaling, which inhibits apoptosis and facilitates HBV replication [26, 27]. Activation of the NF-κB signaling may also restrict HBV transcription and replication. Tumor necrosis factor-α and MYD88 of the interferon signaling pathway have been documented to activate the NF-κB signaling and repress HBV replication [28, 29]. In addition, NF-κB p65 might compete with the transcription factor Sp1 to bind to HBV promoters [30].

In this study, we explored the role of HNF1α in the regulation of HBV gene expression and replication. We demonstrated that besides its well-known activity in stimulating the Sp1 promoter, HNF1α could indirectly inhibit HBV gene expression and replication by activating the NF-κB signaling through increasing p65 expression and protein stability.

## Materials and methods

### Plasmids

pHBV1.3 containing a terminally redundant replication-competent HBV genome (1.3 copy, subtype *adw*) and pCDNA3.1-LHBs-flag were kindly provided by Dr. Jianhua Li (Fudan University, China) [31]. pHBV1.3LHBs- was generated by altering the start codon (ATG to ACG) of the preS1 open reading frame (ORF) in pHBV1.3 using the KOD-Plus mutageneis kit (TOYOBO, Osaka, Japan). pCMV-IκBα-SR was a generous gift from Dr. Muxiang Zhou (Emory University School of Medicine, USA). IκBα-SR is a dominant repressor form of IκBα in which serine 32 is mutated to alanine [32]. To construct pCMV-p65, pCMV-HNF1α, pCMV-HNF4α and pCMV-NTCP, the cDNAs of the respective genes were inserted in pCMV respectively. The HNF1α mutant expression plasmids (pCMV-Nt, pCMV-POU, pCMV-TD, pCMV-delNt, pCMV-delPOU, pCMV-delTD) was constructed by making the corresponding deletions in pCMV-HNF1α. The firefly luciferase reporter constructs of HBV promoters/enhancers (Sp1, Sp2, EnII/Cp, and EnI/Xp) were described previously [33]. The renilla luciferase expression plasmid, pRL-TK, and pGL3-basic-Luciferase, are products of Promega. pNF-κB-Luc is a NF-κB-dependent luciferase reporter plasmid obtained from Stratagene Corporation (La Jolla, CA). For RNA interference of endogenous *HNF1α* expression, a DNA fragment encoding the hairpin RNA (shRNA) (target sequence 5’-CCTTGTTCTGTCACCAATGTA-3’, corresponding to nt2370-2391 of the *HNF1α* reference sequence (NM_000545)) was inserted into pLKO.1 (Addgene plasmid 10879). shRNA against EGFP was similarly constructed.

### Cell culture and transfection

Human hepatoma cell line Huh7 and embryonic kidney cell line HEK293T were cultured in Dulbecco’s modified Eagle’s medium supplemented with 10% fetal bovine serum, 2mM L-glutamine, 100U/ml penicillin and 100 mg/ml streptomycin (GIBCO) and maintained in 5% CO_2_ at 37°C. DNA transfection was performed using Turbofect (Thermo Scientific) following the manufacturer’s protocol. Lentiviruses were harvested at 48 hours after transfection of HEK293T cells per 6 cm dish with 5 μg of pLKO.1-shRNA, 3.75 μg of pMD2.G (Addgene plasmid 12259) and 2.25 μg of psPAX2 (Addgene plasmid 12260). For transduction, appropriate amount of lentivirus-containing supernatant was added to the medium. Polybrene was added to a final concentration of 5 μg/ml to optimize the lentiviral transduction.

### Real-time quantitative PCR (qPCR)

Total cellular RNA was isolated using TRIzol reagent (Invitrogen) and subjected to reverse transcription using the Transcript one-step gDNA removal and cDNA synthesis supermix kits (TakaRa) according to the manufacturer’s instructions. cDNA mixture was used for qPCR with the SYBR Green Realtime PCR Master Mix (TOYOBO) in Eppendorf Mastercycler Real-time PCR system. The relative quantities of mRNAs were calculated based on the comparative Ct method. GAPDH was used for the normalization of mRNA levels.

HBV DNA was extracted from virions in the cell culture supernatant as described previously [31] with modifications. Briefly, 100 μl of culture supernatant was digested with DNase I (Promega) at 37°C for 1 hour to remove plasmid DNA. After inactivation of the enzyme with 10 mM EDTA at 37°C for 30 minutes, the sample was incubated at 37°C overnight with 100 μl lysis buffer (20 mM Tris-HCl, 20 mM EDTA, 50 mM NaCl, and 0.5% sodium dodecyl sulfate) containing 50 μg proteinase K. After incubation, viral DNA was isolated using phenol-chloroform extraction and quantified with real-time PCR with the primer pair (AATGCCCCTATCTTATCAACAC/GAGATTGAGATCTTCTGCGACG). Amplification was performed according to the following protocol: 95°C for 10 min, 40 cycles of 95°C for 10 s, 55°C for 15 s, and 72°C for 20 s. Serial dilutions of a plasmid containing a HBV insert were used as quantification standards.

### Southern blot

HBV DNA replicative intermediates from intracellular core particles were extracted as previously described [31], electrophoresed, and transferred onto nylon membrane (GE Healthcare). A digoxigenin (DIG)-labeled RNA probe specific to the core ORF was prepared with the DIG Northern starter kit (Roche Diagnostics) and the hybridization performed according to the manufacturer’s instruction.

### Northern blot

10 μg of total RNA was subject to formaldehyde-1% agarose gel and transferred onto nylon membrane. HBV RNA transcripts were detected with a DIG-labeled HBV RNA probe prepared using the DIG Northern starter kit (Roche Diagnostics) according to the manufacturer’s instruction. 1 μg of total RNA was taken to run an agarose gel to detect the 28S and 18S rRNAs as loading controls.

### HBsAg and HBeAg measurements

48 hours post transfection, cell culture supernatants were collected and cells were lysed with 100 μl of RIPA lysis buffer (25 mM Tris, pH 7.4, 150 mM NaCl, 1% NP-40, 1% sodium deoxycholate, 0.1% SDS). HBsAg and HBeAg were detected using a commercial enzyme-linked immunosorbent assay (ELISA) kits (Kehua, Shanghai, China).

### Isolation of cytoplasmic and nuclear proteins

Two days post-transfection, cells were washed with PBS three times and the cytoplasmic and nuclear protein fractions were separated and extracted with Nuclear/Cytosol Fractionation Kit (Sangon Biotech, Shanghai, China) according the manufacturer’s instruction.

### Western blot

Cells were lysed with the RIPA lysis buffer. The cell lysate was subject to 10% SDS-polyacrylamide gel electrophoresis and proteins were transferred onto polyvinylidene difluoride (PVDF) membrane (Roche Diagnostics). The membrane was blocked with 5% blotting milk in PBST (20 mM Tris-HCl pH7.6, 137 mM NaCl, 0.5% Tween) and then incubated with one of the primary antibodies [anti-HNF1α (sc-6548, Santa Cruz), anti-HNF4α (C11F12, Cell Signaling Technology), anti-p65 (sc-372, Santa Cruz), anti-HDAC2 (sc-5549, Santa Cruz), anti-preS1 (sc57761, Santa Cruz), anti-flag (Sigma), and anti-β-actin (Sigma)] overnight at 4°C. The membrane was washed with PBST and incubated with the horseradish peroxidase (HRP)-labeled secondary antibody (Sigma) at room temperature for 2 hours. Signals were visualized with enhanced chemiluminescence detection reagents (Amersham).

### Luciferase assays

Luciferase assays were performed using the Dual-Luciferase Reporter System Detection kit (Promega). 48 hours post transfection, cells were washed with PBS and lysed. The cell lysate was centrifuged to remove cellular debris. 10 μl of the cleared lysate was used for the simultaneous detection of firefly (Fluc) and renilla (Rluc) luciferases with a Luminometer (Promega). Reporter values were normalized and presented as the ratio of Fluc/Rluc.

### Statistical analysis

Results are presented as means with standard errors of the means (SEM) calculated from at least three independent repeats. Statistical significance is examined using student’s *t*-test. A *p*-value <0.05 was considered statistically significant in all analyses. GraphPad 6 was used for plotting and statistical analysis.

## Results

### HNF1α inhibits HBV gene expression and replication in Huh7 cells

**Fig 1:**
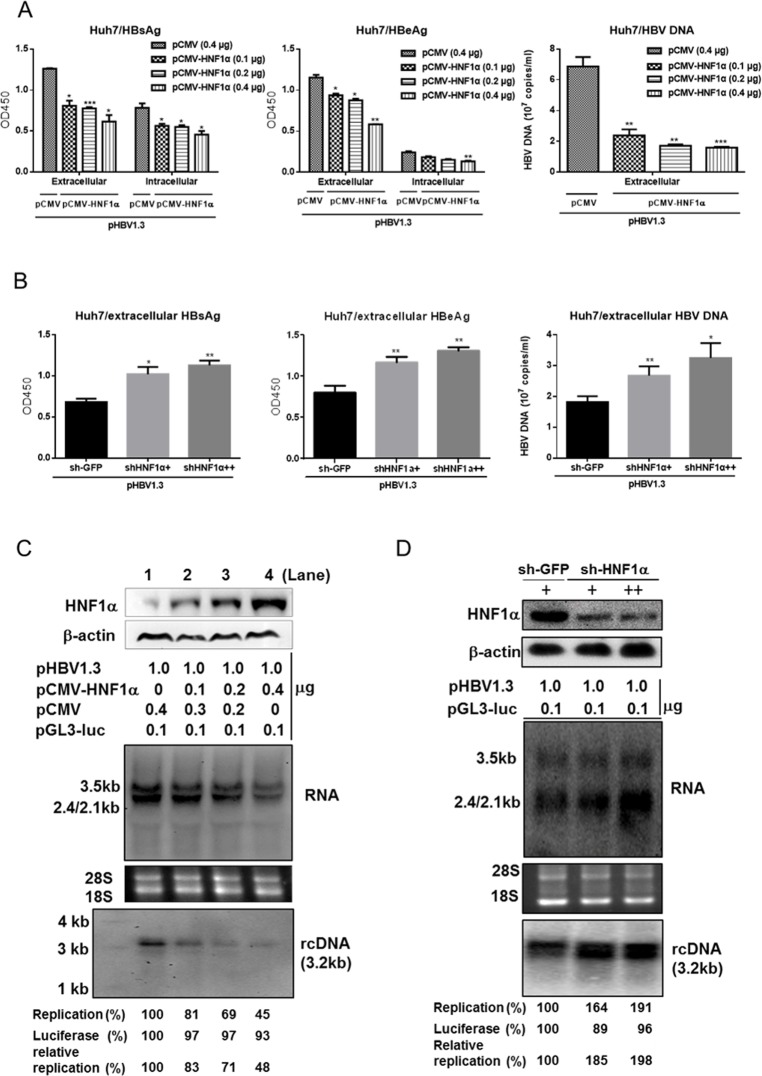
HNF1α inhibits HBV gene expression and replication in Huh7 cells. (A) *HNF1α* overexpression inhibited HBV antigen and DNA productions. Huh7 cells cultured in 24-well plate were co-transfected with the indicated plasmids. 48 hours post-transfection, the extracellular levels of HBsAg, HBeAg and HBV DNA and the intracellular levels of HBsAg and HBeAg were measured. (B) Knockdown of endogenous *HNF1α* expression enhanced HBV antigen and DNA productions. Huh7 cells cultured in 6-well plate were transduced by the lentivirus expressing sh-HNF1α (+ and ++ stand for 0.5 ml and 1 ml lentivirus supernatant per well, respectively) or sh-EGFP. 16 hours post-transduction, cells were transfected with pHBV1.3 (1 μg). The extracellular levels of HBsAg, HBeAg and HBV DNA were determined. Means and SEMs of data from at least three independent tests were plotted. * *P* < 0.05, ** *P* < 0.01, *** *P* < 0.001. Intracellular core-associated HBV DNA and viral mRNAs from *HNF1α*-overexpressing (C) or *HNF1α* expression knockdown (D) Huh7 cells were examined using Southern blot (*upper*) and Northern blot (*lower*), respectively. HNF1α protein expression was confirmed with Western blot. HBV replication intermediates were quantified using densitometry scanning. Transfection efficiency was normalized by using co-transfected pGL3-luciferase plasmid and control measurements were taken as 100%.

To explore the role of HNF1α in regulating HBV gene expression and replication, we first transfected Huh7 cells with pHBV1.3 and an increasing amount of pCMV-HNF1α. Western blot results confirmed a dose-dependent increase in the expression of HNF1α protein (Fig 1C). Both the extracellular and intracellular levels of HBsAg and HBeAg declined upon exogenic *HNF1α* expression (Fig 1A). A similar pattern was observed with the extracellular viral DNA (Fig 1A). HBV replication intermediates and mRNAs were further examined with Southern blot and Northern blot respectively. As *HNF1α* expression was increased, the levels of 3.2kb rcDNA, 3.5kb and 2.4kb/2.1kb transcripts diminished (Fig 1C). In contrast, HBV gene expression and replication were elevated in Huh7 cells with reduced endogenous *HNF1α* expression caused by the infection of the anti-*HNF1α* shRNA-expressing lentivirus (Fig 1B and 1D). It is notworthy that there was no apparent change in the level of HNF1α protein in Huh7 cells transfected with pHBV1.3 (S1 Fig). We also performed overexpression of HNF4α and NTCP in Huh7 cells. Overexpression of HNF4α, the well-established positive regulator of HBV gene expression, upregulated HBsAg and HBeAg levels, while overexpression of the HBV receptor NTCP [34] had no effect (S2 Fig). Moreover, the inhibition of HBV gene expression and replication by the overexpression of HNF1α was also observed in HepG2 cells (S3 Fig). These results indicate that HNF1α specifically inhibits HBV gene expression and replication in Huh7 and HepG2 cells.

### HNF1α’s inhibition of HBV gene expression is independent of LHBs

**Fig 2:**
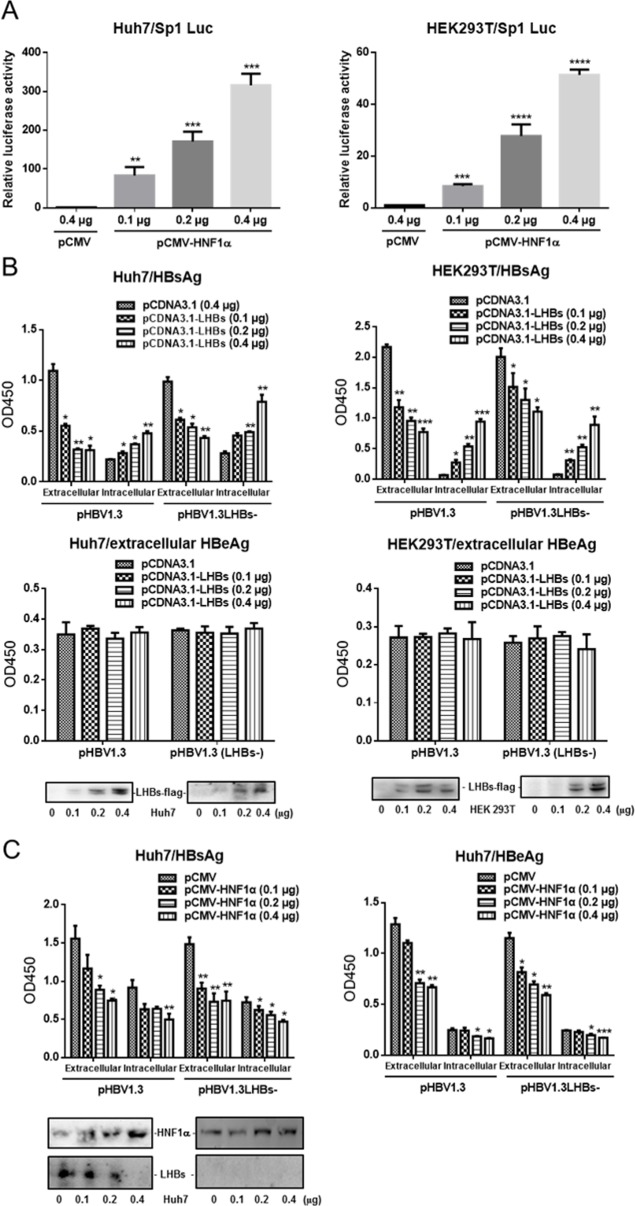
HNF1α’s inhibition of HBV gene expression is independent of LHBs. (A) Huh7 (*left*) and HEK293T cells (*right*) were cultured in 24-well plate and co-transfected with the Sp1 reporter plasmid, pRL-TK and pCMV-HNF1α or pCMV. Means and SEMs of relative luciferase activity data are plotted, with the means of the values from pCMV-transfected cells taken as 1. (B) Huh7 (*left*) and HEK293T cells (*right*) were cultured in 24-well plate and co-transfected with the indicated plasmids. The extracellular and intracellular levels of HBsAg and HBeAg were determined. The expression levels of Flag-tagged LHBs were checked with Western blot analysis. (C) Huh7 cells were cultured in 24-well plate and co-transfected with the indicated plasmids. The extracellular and intracellular levels of HBsAg and HBeAg were determined. The virus-derived LHBs was checked with Western blot analysis. Means and SEMs of data from at least three independent tests were plotted. * P <0.*05*, ** P <0.*01*, *** P <0.*001*, **** P <0.*0001*.

Since large amount of LHBs can cause the retention of viral particles in ER [12, 14], we wondered if HNF1α’s inhibition of HBV gene expression was mediated through LHBs. Western blot results showed that HNF1α was expressed in Huh7 cells but not HEK293T cells (data not shown). To confirm the activation of the Sp1 promoter by HNF1α, Huh7 or HEK293T cells were co-transfected with pCMV-HNF1α and the Sp1 reporter plasmid. Luciferase assay results clearly showed that HNF1α activated the Sp1 promoter in both Huh7 and HEK293T cells (Fig 2A), which is consistent with previous reports.

To test whether LHBs is capable of repressing HBV antigen expression, Huh7 or HEK293T cells were co-transfected with pHBV1.3 or pHBV1.3LHBs- together with an increasing amount of pCDNA3.1-LHBs. pHBV1.3LHBs- is deficient in LHBs expression due to the mutation of the start codon of preS1. As shown in Fig 2B, pCDNA3.1-LHBs and pHBV1.3 co-transfected cells displayed a reduction in extracellular HBsAg and an increase in intracellular HBsAg, consistent with the notion that overexpression of LHBs leads to cellular retention of viral particles. No notable change in the level of HBeAg was observed in pCDNA3.1-LHBs co-transfectants. Similar results were obtained with the cells co-transfected with pHBV1.3LHBs- and pCDNA3.1-LHBs. Next, Huh7 cells were co-transfected with pHBV1.3 or pHBV1.3LHBs- together with an increasing amount of pCDNA3.1-HNF1α. As shown in Fig 2C, both the extracellular and intracellular levels of HBsAg and HBeAg, especially for LHBs expression, declined upon *HNF1α* overexpression, regardless of whether the cells were co-transfected with pHBV1.3 or pHBV1.3LHBs-. Apparently, the effects of LHBs on HBV antigen expression were different from those caused by *HNF1α* overexpression.

### HNF1α activates the NF-κB signaling

**Fig 3:**
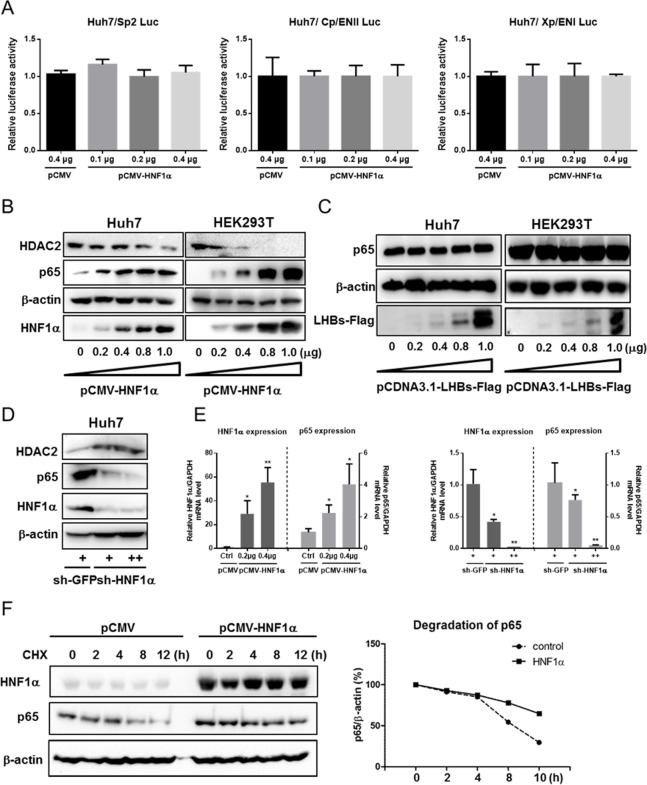
HNF1α promotes p65 expression and protein stability. (A) Huh7 cells were cultured in 24-well plate and co-transfected with the Sp2, ENII/Cp or ENI/Xp reporter plasmid, pRL-TK and pCMV-HNF1α or pCMV. Means and SEMs of relative luciferase activity data are plotted, with the means of the values from pCMV-transfected cells taken as 1. (B) Western blot analysis of the p65 and HDAC2 levels upon overexpression of HNF1α. Huh7 cells were cultured in 24-well plate and co-transfected with pCMV-HNF1α. (C) Western blot analysis of the p65 level upon overexpression of LHBs. (D) Western blot analysis of p65, and HDAC2 levels in Huh7 cells transduced with the lentivirus expressing sh-HNF1α (+ and ++ stand for 0.2 ml and 0.4 ml lentivirus supernatant per well, respectively) or sh-EGFP. (E) The mRNA expression of *HNF1α* and *p65* were determined with real-time qPCR and normalized against GAPDH. Huh7 cells transfected with pCMV-HNF1α or pCMV (*left*) or transduced with the lentivirus expressing sh-HNF1α or sh-EGFP. Means and SEMs of data from three independent experiments are plotted with the value of the control takens as 1. *P < 0.*05*, **P < 0.*01*. (F) Huh7 cells (1x10^6^) cultured in 12-well plate were transfected with 0.5 μg of pCMV-HNF1α. After treatment with cycloheximide (CHX) for different time intervals, Western blot was performed on cell lysate to detect p65. Protein bands were scanned, quantified and normalized against β-actin. Each time point represents the relative degradation efficiency (%) versus CHX treatment group of 0 hour time point.

Luciferase assay results showed that the activities of other HBV promoters/enhancers (Sp2, Cp/EnII, Xp/EnI) were not altered by HNF1α overexpression (Fig 3A), though they could be activated by HNF4α overexpression (S4 Fig). Thus it is possible that HNF1α inhibits HBV gene expression indirectly.

Given that NF-κB is known to repress HBV replication, we wondered whether HNF1α might exert its anti-HBV effect by activating the NF-κB signaling. To test this hypothesis, Huh7 and HEK293T cells were co-transfected with an increasing amount of pCMV-HNF1α. Western blot results showed that the level of NF-κB p65 rose along with HNF1α in a dose-dependent manner in Huh7 and HEK293T cells (Fig 3B). Meanwhile, the level of histone deacetylase 2 (HDAC2), a downregulator of NF-κB activity [35–37] decreased upon *HNF1α* overexpression (Fig 3B). In contrast, knockdown of the endogenous *HNF1α* expression was accompanied by a decrease in the level of p65 and an increase in HDAC2 (Fig 3D). There was no change in the level of p65 when LHBs was overexpressed in Huh7 and HEK293T cells (Fig 3C). Furthermore, there was no apparent decrease in the level of the positive regulator HNF4α in Huh7 cells with the overexpression of HNF1α or p65 (S5 Fig). Therefore, under the current experiment conditions, the HNF1α-mediated inhibitory effects on HBV gene exprerssion were not due to the downregulation of HNF4α.

Moreover, *HNF1α* overexpression could increase the level of *p65* mRNA by 2–3 fold while knockdown of the endogenous *HNF1α* expression resulted in a sharp decrease in the *p65* mRNA expression (Fig 3E). To assess the possible effect of HNF1α on the protein stability of p65, Huh7 cells were transfected with pCMV-HNF1α or the vector pCMV, followed by the treatment with 50 μg/ml of cycloheximide (CHX), an inhibitor of protein synthesis, for different durations (0, 2, 4, 8, 12 hours). Western blot results showed that the degradation of p65 was slower in HNF1α-overexpressing cells (Fig 3F). The difference between relative p65 protein levels without or with HNF1α overexpression was only apparent after longer (>8 hours) CHX treatments may suggest that under the experiment conditions we use, this effect of HNF1α was fairly limited and required a long time to accumulate a measurable difference.

**Fig 4:**
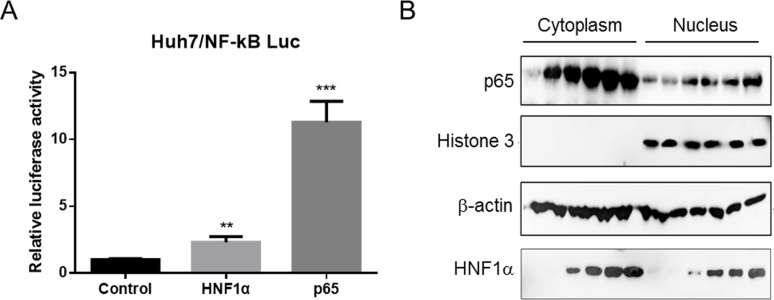
HNF1α activates the NF-κB signaling. (A) Huh7 cells were co-transfected with pNF-κB-luc (0.2 μg), pRL-TK (0.1 μg) and pCMV-HNF1α (0.2 μg) or pCMV-p65 (0.2 μg). Means and SEMs of relative luciferase activity data are plotted, with the means of the values from pCMV-transfected cells (control) taken as 1. ** P < 0.*01*, *** P < 0.*001*. (B) Huh7 cells were co-transfection with pCMV-HNF1α 0.1, 0.2, 0.4, 0.8, or 1.0 μg. Cells were lysed and separated into cytoplasmic and nuclear fractions. Histone 3 served as a loading control of nuclear fraction samples.

To examine the effect of HNF1α on the NF-κB signaling, Huh7 cells were co-transfected with the NF-κB-dependent luciferase reporter and pCMV-HNF1α. HNF1α modestly activated the NF-κB-dependent reporter activity (2–3 fold) (Fig 4A). Western blot results showed that as HNF1α expression was increased, the level of p65 was elevated in both the cytoplasm and nucleus of Huh7 cells (Fig 4B). Since nuclear accumulation of p65 is a hallmark of an activated NF-κB signaling, these results indicate that HNF1α can activates the NF-κB signaling.

### HNF1α’s anti-HBV effects require the activation of the NF-κB signaling

**Fig 5:**
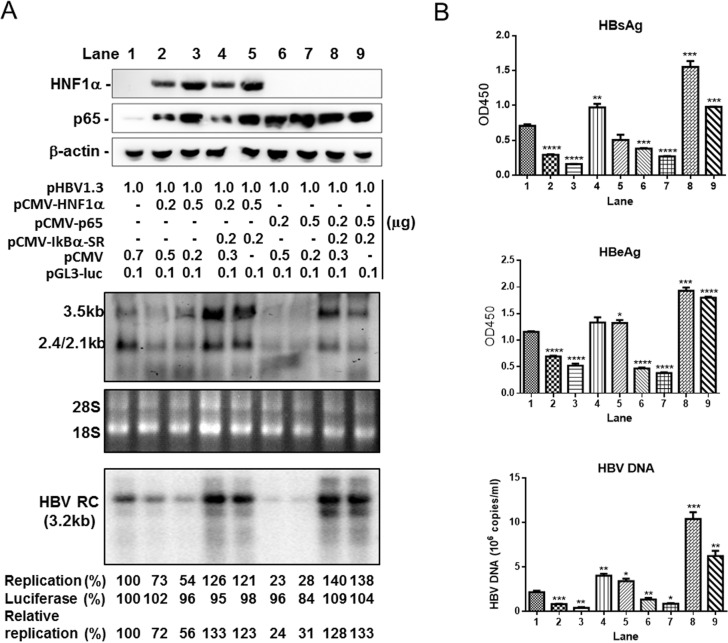
HNF1α inhibits HBV gene expression and replication through activating the NF-κB signaling. Huh7 cells cultured in 6-well plate were co-transfected with the indicated amount of the plasmids. (A) The replication intermediates in viral core particles were examined with Southern blot and Viral RNAs with Northern blot. RC, relaxed circular DNA. 18S/28S RNAs served as the RNA loading control. HBV replication intermediates were quantified using densitometry scanning. Transfection efficiency was normalized by using co-transfected pGL3-luciferase plasmid and control measurements were taken as 100%. (B) The culture supernatants were collected to detect HBsAg, HBeAg and HBV DNA. Means and SEMs of data from three independent experiments are plotted. * P <0.*05*, ** P <0.*01*, *** P <0.*001*, **** P < 0.*0001*.

To ascertain whether HNF1α inhibits HBV gene expression and replication through activating the NF-κB signaling, Huh7 cells were co-transfected with pHBV1.3, pCMV-HNF1α or pCMV-p65, with or without pCMV-IκBα-SR that encodes a dominant repressor of the NF-κB signaling. The results of Southern blot and Northern blot showed that the levels of both viral rcDNA and mRNAs decreased upon *p65* overexpression (Fig 5A, lanes 6 & 7). Concurrently, the levels of HBsAg, HBeAg and HBV DNA also declined (Fig 5B). IκBα-SR could abrogate this repression by p65 (Fig 5A, lanes 8 & 9 and Fig 5B). A similar pattern was observed with pCMV-HNF1α-co-transfected cells. IκBα-SR completely counteracted HNF1α's inhibition of HBV gene expression and replication (Fig 5A, lanes 4 & 5 and Fig 5B). These results suggest that HNF1α most likely inhibits HBV gene expression and replication by activating the NF-κB signaling.

### All the domains of HNF1α are required for HNF1α's inhibition of HBV gene expression

**Fig 6:**
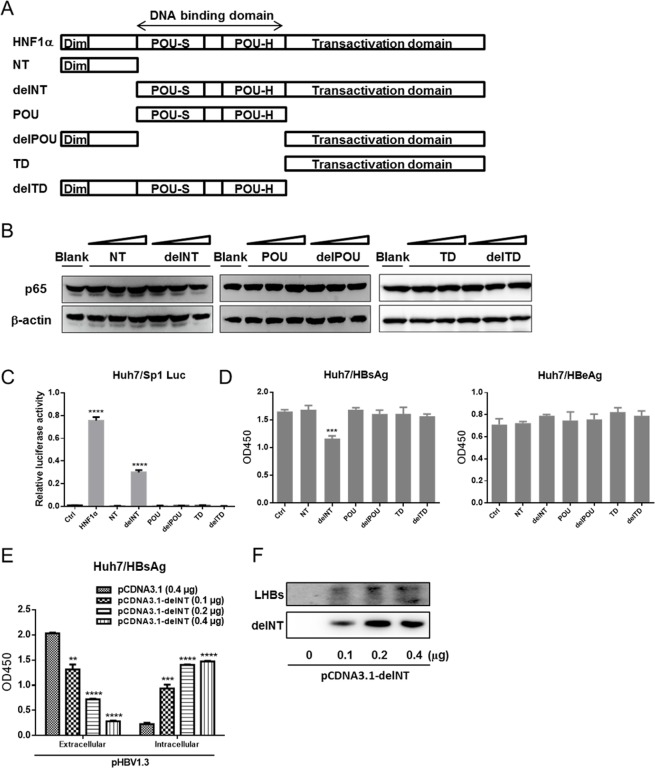
Domains of HNF1α required for HNF1α's inhibition of HBV gene expression. (A) Schematic presentation of HNF1α domains and mutants. (B) Western blot analysis of the p65 level in Huh7 cells transfected with the constructs encoding HNF1α mutants. (C) Huh7 cells were cultured in 24-well plate and co-transfected with the Sp1 reporter plasmid (0.2 μg), pRL-TK (0.1 μg) and pCMV-HNF1α wid-type or mutant (0.2 μg) or pCMV (0.2 μg). Means and SEMs of relative luciferase activity data are plotted, with the means of the values from pCMV-HNF1α-transfected cells taken as 1. (D) Huh7 cells were cultured in 24-well plate and co-transfected with pHBV1.3 (0.2 μg) and each construct of HNF1α mutant (0.2 μg). HBsAg and HBeAg were determined. (E) Huh7 cells were co-transfected with pHBV1.3 (0.2 μg) and an increasing amount of pCMV-delNT (0.1, 0.2, 0.4 μg) in 24-well culture plate. HBsAg and HBeAg were measured. (F) Western blot analysis of the expression levels of LHBs and delNT mutant. Means and SEMs of data from three independent experiments are plotted. **P < 0.*01*, *** P < 0.*001*, ****P < 0.*0001*.

To define the domain(s) of HNF1α involved in mediating the upregulation of *p65*, a series of GFP-tagged HNF1α mutants with one or more domain(s) deleted were created (Fig 6A). Each mutant was expressed in Huh7 cells. Western blot results showed that none of the mutants could enhance the level of p65 as did the wild type HNF1α (Fig 6B). Luciferase assay results revealed that only the delNT mutant could activate the Sp1 promoter (Fig 6C).

Next, Huh7 cells were co-transfected with pHBV1.3 and the expression plasmid for each HNF1α mutant. Only the delNT mutant could reduce the extracellular level of HBsAg while the other mutants were ineffective in altering the level of HBsAg. In addition, none of the mutants had any effect on the level of HBeAg (Fig 6D). Furthermore, as shown in Fig 6E and Fig 6F, the delNT mutant could increase the expression of LHBs in Huh7 cells and the intracellular level of HBsAg. In other words, the effect of overexpression of the delNT mutant was similar to the overexpression of LHBs (Fig 2B). These data further prove that the mechanisms of HNF1α-mediated inhibition of HBV gene expression and HNF1α's modulation of LHBs expression are very different. The former requires all the domains of HNF1α while the latter can be achieved without the N-terminal dimerization domain.

## Discussion

HNF1α as a key member of the liver enriched transcription factor family collaborates with other family factors to participate in a wide range of hepatocellular biochemical processes including lipid metabolism, gluconeogenesis and deoxygenation of xenobiotics. The role of HNF1α in the regulation of HBV replication is not fully understood. HNF1α is required to induce the expression of LHBs *in vitro* by binding to and activate viral Sp1 promoter [13]. However, there is no obvious increase in the preS1 mRNA level in HNF1α-null HBV transgenic mice compared to normal HBV transgenic mice [19]. Both positive and negative regulations of HBV replication have been documented for HNF1α [12–17]. In this study, we found that *HNF1α* overexpression downregulates HBV gene expression and replication in Huh7 cells while *HNF1α*-knockdown facilitates HBV transcription and replication. HNF1α can promote the expression of p65 protein via up-regulating *p65* mRNA transcription and p65 protein stability, resulting in the activation of the NF-κB signaling that in turn inhibits HBV gene expression and replication. Our findings may help explain the puzzling observation in HNF1α-null HBV transgenic mice wherein the HBV replication intermediates in hepatocytes were increased and cccDNA more readily detected [19].

In our study, HNF1α strongly activates the Sp1 promoter and LHBs inhibits the secretion of HBsAg, which is consistent with the previous reports [12–14]. However, we found that overexpression of *HNF1α* in Huh7 cells reduced not only the extracellular level but also the intracellular level of HBsAg, plus those of HBeAg. Further examination revealed that the HBV transcription was also downregulated by *HNF1α* overexpression in Huh7 cells. Knockdown of the endogenous *HNF1α* expression resulted in opposite effects. Thus, LHBs is unlikely to mediate HNF1α’s inhibition of HBV gene expression and replication. Actually, the decrease in HBV RNA expression upon *HNF1α* overexpression is intriguing, since the activities of Sp2, Cp/EnII and Xp/EnI are not affected by HNF1α (Fig 3A). One possible explanation is that the HNF1α/p65 signaling has a negative effect on the activities of HBV promoters/enhancers only when these *cis*-elements are located in the context of HBV genome. Another possibility is that the HNF1α/p65 signaling downregulates HBV gene expression post-transcriptionally. TNF-α-activated NF-κB has been reported to inhibit HBV replication by interfering with HBV capsid formation [25, 27]. In our study, the reduction in the DNA intermediates upon *HNF1α* overexpression might be partly owing to this mechanism. However, viral mRNA expression in transient replicon transfection cell culture system is in large part derived from the transfected HBV replicon. It is unlikely that NF-κB can downregulate HBV mRNA expression by disrupting nucleocapsids in this system. Therefore, we propose that other post-transcriptional mechanisms are at work, which warrants future exploration.

The current study raises the question of how HNF1α stimulates the NF-κB signaling. Our results suggest that there might be several possible mechanisms working at different levels. First, though moderate, there is an increase of *p65* mRNA expression upon *HNF1α* overexpression. Second, HNF1α improves the stability of p65 protein. Third, the HDAC2 level diminished upon *HNF1α* overexpression releases HDAC2’s negative regulation of p65 activity. HDAC2 has been reported to interact with HDAC1 and thereby indirectly associate with p65 to inhibit the transcriptional activation by p65 [35–37]. Therefore, the reduction in the HDAC2 expression would promote the transcriptional activity of p65. In addition, *HNF1α* overexpression in Huh7 cells may also promote the nuclear accumulation of p65, probably as a result of the first two mechanisms stated above.

None of the HNF1α mutants in this study is able to increase the p65 expression or has a negative effect on HBeAg expression. Only the delNT mutant has regulatory effects on HBsAg secretion. Interestingly, the delNT mutant that lacks the N-terminal dimerization domain strongly activates the Sp1 promoter and acts similarly to LHBs. These results suggest that the structural integrity of HNF1α is important for the upregulation of p65 and downregulation of HBV gene expression. On the other hand, LHBs, though its promoter (Sp1) is induced by HNF1α, does not play a role in regulating p65 activity and HBV replication.

In conclusion, our findings indicate that on one hand, HNF1α can modulate the expression of HBV LHBs to interfere virion production. On the other hand, HNF1α is capable of limiting HBV transcription and replication by activating the NF-κB signaling. Whether this HNF1α-mediated restriction of HBV transcription and replication is beneficial to the chronic infection of HBV is an open question to be investigated in the future.

## Supporting information

S1 Fig HBV gene expression does not affect HNF1α protein expression in Huh7 cells. Huh7 cells cultured in 24-well plate were transfected with an increasing amount of pHBV1.3 (0.1, 0.2, 0.3, 0.4 μg). 48 hours post-transfection, the supernatants were collected for HBV ELISA tests (HBsAg, HBeAg) and cell lysates for Western blot detection of HNF1α and β-actin proteins.
(TIF)

S2 Fig Overexpression of HNF4α upregulates HBsAg and HBeAg levels. Huh7 cells cultured in 24-well plate were co-transfected with pHBV1.3 (0.2 μg) and an increasing amount of pCMV-HNF4α or pCMV-NTCP (0.1, 0.2, 0.4 μg). HBsAg and HBeAg were measured. The expression levels of HNF4α, NTCP and β-actin were determined using Western blot. Means and SEMs of data from three independent experiments are plotted. *P < 0.*05*, **P < 0.*01*.
(TIF)

S3 Fig HNF1α inhibits HBV gene expression and replication in HepG2 cells. HepG2 cells cultured in 6-well plate were co-transfected with the indicated amount of the plasmids. (A) The culture supernatants were collected for the measurements of HBsAg, HBeAg and HBV DNA. (B) The replication intermediates in viral core particles were examined with Southern blot and viral RNAs with Northern blot. RC, relaxed circular DNA. 18S/28S RNAs served as the RNA loading control. HBV replication intermediates were quantified using densitometry scanning. Transfection efficiency was normalized by using co-transfected pGL3-luciferase plasmid and control measurements were taken as 100%. Means and SEMs of data from three independent experiments are plotted. * *P* <0.05, ** *P* <0.01.
(TIF)

S4 Fig Activation of the HBV promoters and enhancers by HNF4α. Huh7 cells cultured in 24-well plate were co-transfected with the indicated HBV promoter/enhancers reporter plasmid (Sp1, Sp2, Cp/ENII, Xp/ENI), pRL-TK and pCMV-HNF4α or pCMV. Means and SEMs of relative luciferase activity data are plotted, with the means of the values from pCMV-transfected cells taken as 1. ** P <0.*01*, **** P < 0.*0001*.
(TIF)

S5 Fig HNF1α or p65 overexpression does not decrease HNF4α protein expression. Huh7 cells cultured in 24-well plate were transfected with pCMV-HNF1α or pCMV-p65 (0.2, 0.4, 0.8, 1.0 μg). The expression of the indicated protein was determined using Western blot 48 hours post-transfection.
(TIF)
