# Supplementary figures and images for: Hepatocyte nuclear factor 1α downregulates HBV gene expression and replication by activating the NF-κB signaling pathway
Source: PLoS One. 2017 Mar 20;12(3):e0174017. doi: 10.1371/journal.pone.0174017 (PMC5358864; doi:10.1371/journal.pone.0174017)

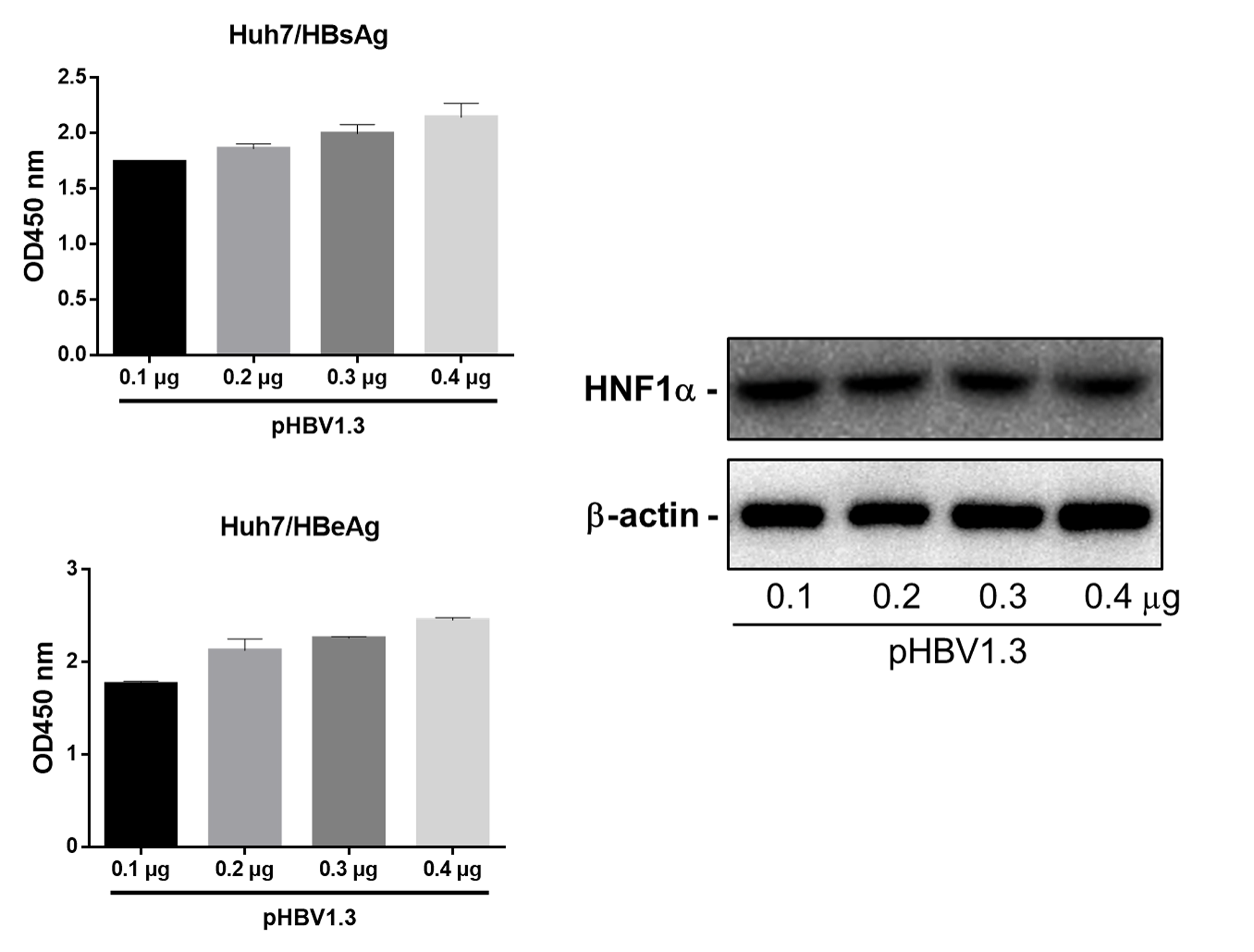

Supplement: S1 Fig — Huh7 cells cultured in 24-well plate were transfected with an increasing amount of pHBV1.3 (0.1, 0.2, 0.3, 0.4 μg). 48 hours post-transfection, the supernatants were collected for HBV ELISA tests (HBsAg, HBeAg) and cell lysates for Western blot detection of HNF1α and β-actin proteins. (TIF) [file pone.0174017.s001.tif]

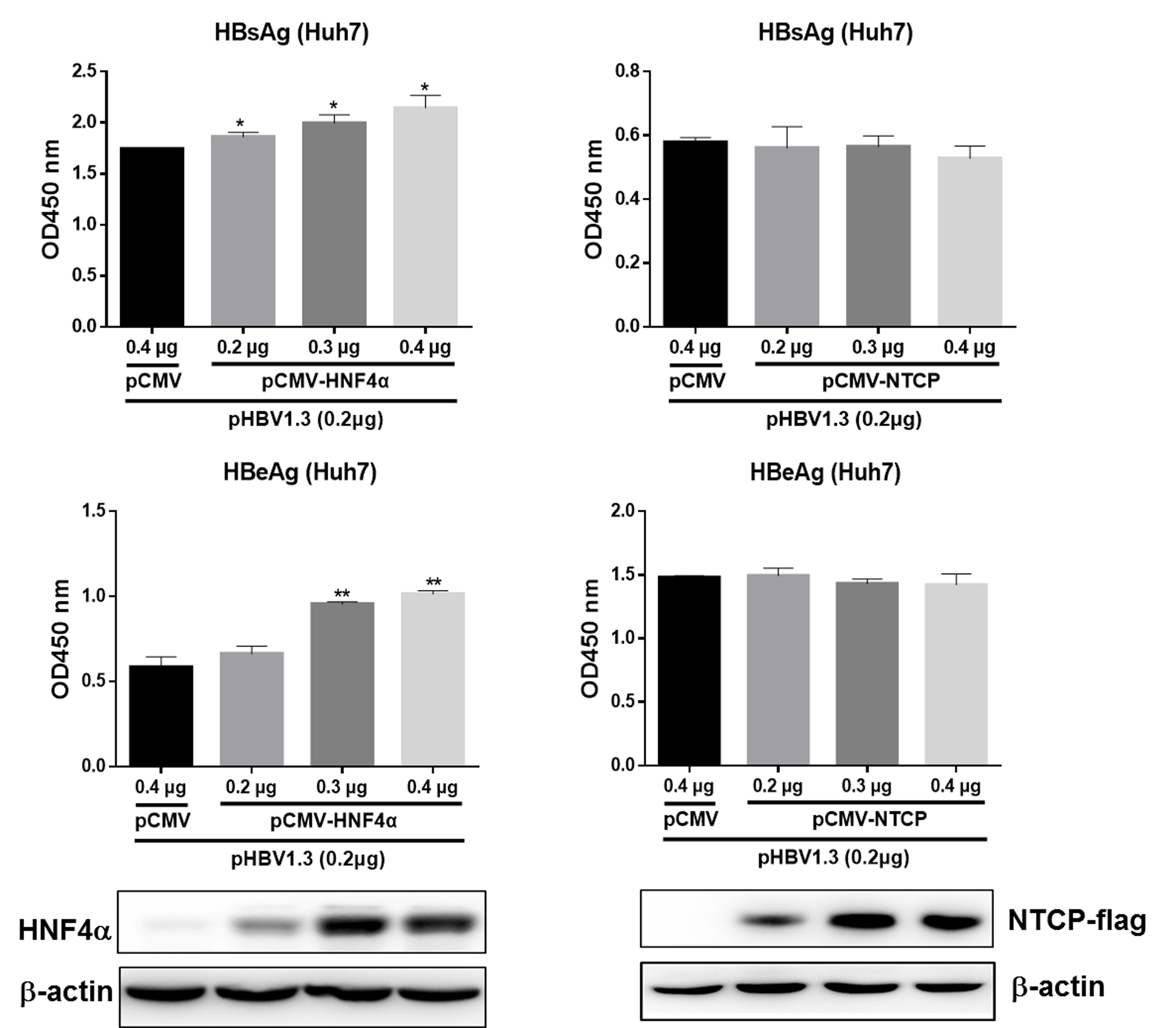

Supplement: S2 Fig — Huh7 cells cultured in 24-well plate were co-transfected with pHBV1.3 (0.2 μg) and an increasing amount of pCMV-HNF4α or pCMV-NTCP (0.1, 0.2, 0.4 μg). HBsAg and HBeAg were measured. The expression levels of HNF4α, NTCP and β-actin were determined using Western blot. Means and SEMs of data from three independent experiments are plotted. *P < 0.05, **P < 0.01. (TIF) [file pone.0174017.s002.tif]

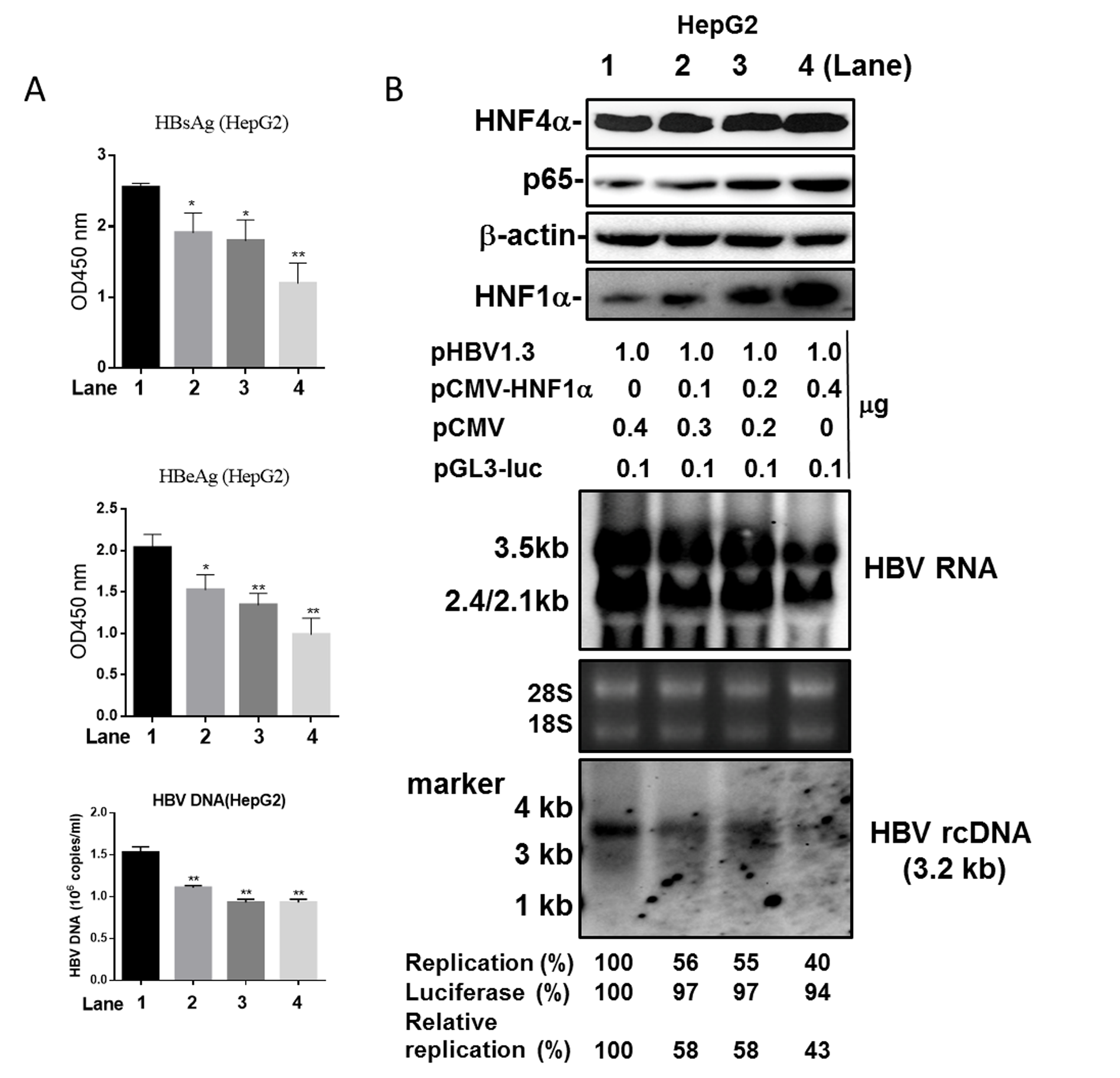

Supplement: S3 Fig — HepG2 cells cultured in 6-well plate were co-transfected with the indicated amount of the plasmids. (A) The culture supernatants were collected for the measurements of HBsAg, HBeAg and HBV DNA. (B) The replication intermediates in viral core particles were examined with Southern blot and viral RNAs with Northern blot. RC, relaxed circular DNA. 18S/28S RNAs served as the RNA loading control. HBV replication intermediates were quantified using densitometry scanning. Transfection efficiency was normalized by using co-transfected pGL3-luciferase plasmid and control measurements were taken as 100%. Means and SEMs of data from three independent experiments are plotted. * P <0.05, ** P <0.01. (TIF) [file pone.0174017.s003.tif]

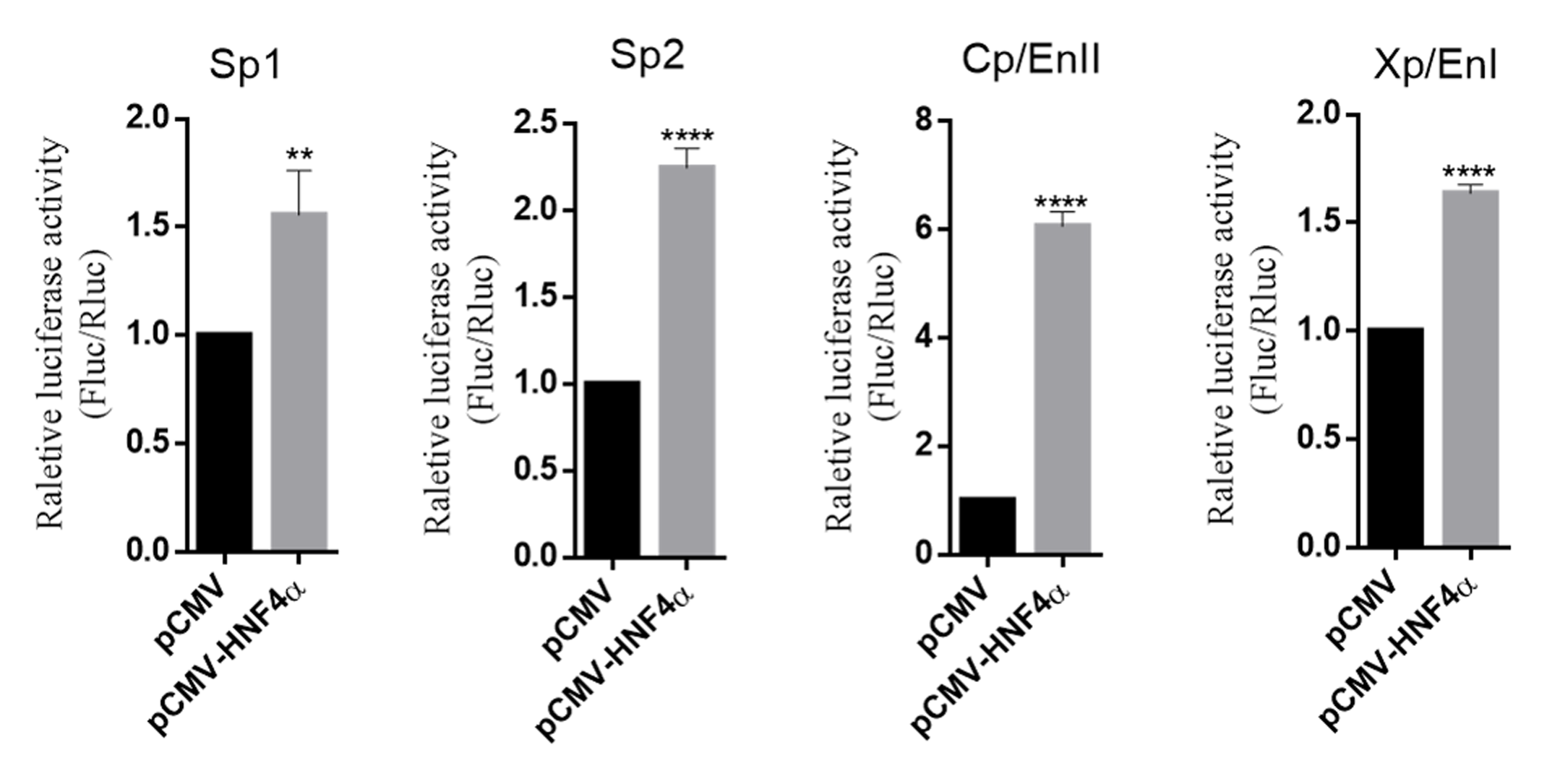

Supplement: S4 Fig — Huh7 cells cultured in 24-well plate were co-transfected with the indicated HBV promoter/enhancers reporter plasmid (Sp1, Sp2, Cp/ENII, Xp/ENI), pRL-TK and pCMV-HNF4α or pCMV. Means and SEMs of relative luciferase activity data are plotted, with the means of the values from pCMV-transfected cells taken as 1. ** P <0.01, **** P < 0.0001. (TIF) [file pone.0174017.s004.tif]

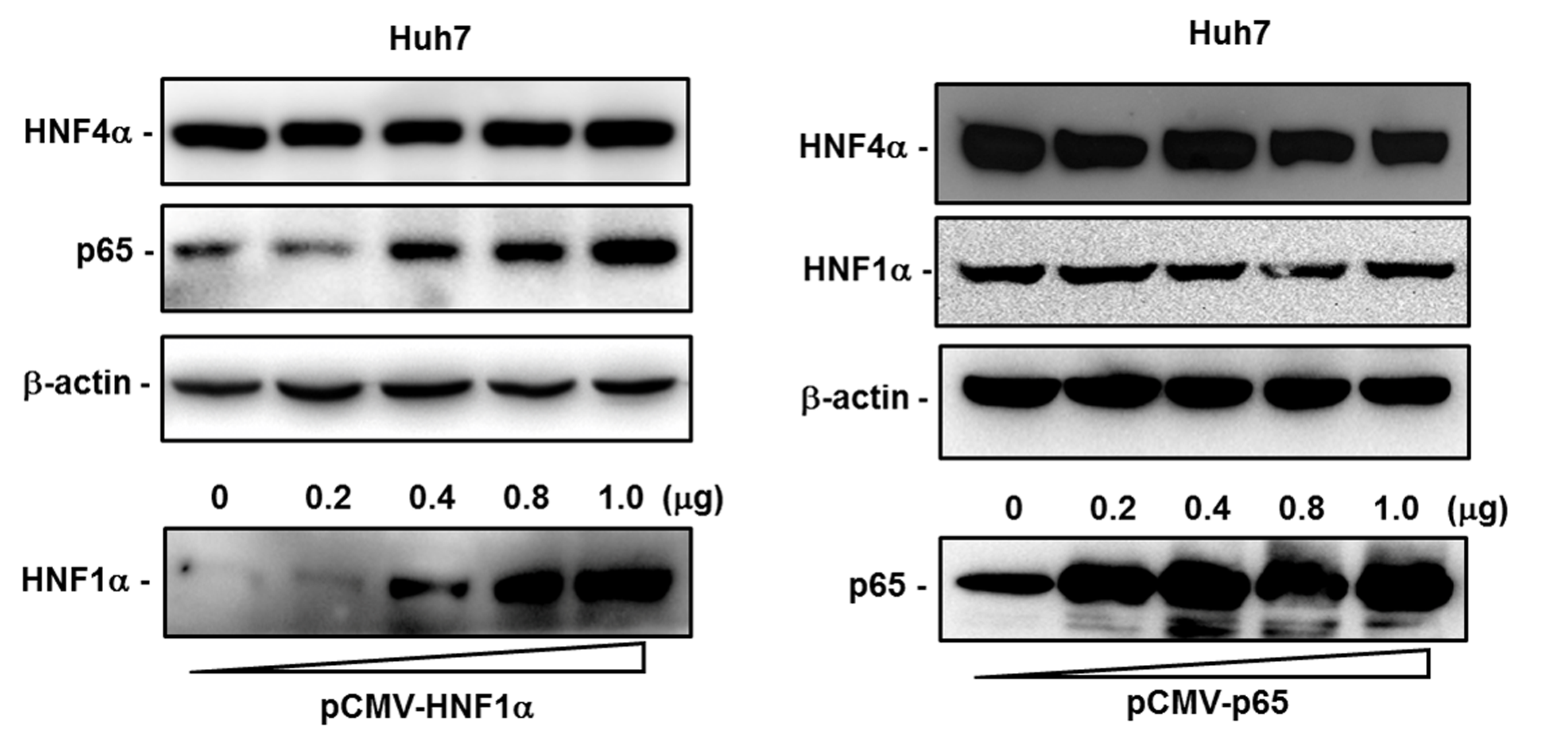

Supplement: S5 Fig — Huh7 cells cultured in 24-well plate were transfected with pCMV-HNF1α or pCMV-p65 (0.2, 0.4, 0.8, 1.0 μg). The expression of the indicated protein was determined using Western blot 48 hours post-transfection. (TIF) [file pone.0174017.s005.tif]
